# Elevated miR-499 Levels Blunt the Cardiac Stress Response

**DOI:** 10.1371/journal.pone.0019481

**Published:** 2011-05-09

**Authors:** Joseph T. C. Shieh, Yu Huang, Jacqueline Gilmore, Deepak Srivastava

**Affiliations:** 1 Gladstone Institute of Cardiovascular Disease, San Francisco, California, United States of America; 2 Division of Medical Genetics, Department of Pediatrics, University of California San Francisco, San Francisco, California, United States of America; 3 School of Medicine, University of California San Francisco, San Francisco, California, United States of America; 4 Division of Pediatric Cardiology, Department of Pediatrics, University of California San Francisco, San Francisco, California, United States of America; 5 Department of Biochemistry and Biophysics, University of California San Francisco, San Francisco, California, United States of America; Keio University, Japan

## Abstract

**Background:**

The heart responds to myriad stresses by well-described transcriptional responses that involve long-term changes in gene expression as well as more immediate, transient adaptations. MicroRNAs quantitatively regulate mRNAs and thus may affect the cardiac transcriptional output and cardiac function. Here we investigate miR-499, a microRNA embedded within a ventricular-specific myosin heavy chain gene, which is expressed in heart and skeletal muscle.

**Methodology/Principal Findings:**

We assessed miR-499 expression in human tissue to confirm its potential relevance to human cardiac gene regulation. Using a transgenic mouse model, we found that elevated miR-499 levels caused cellular hypertrophy and cardiac dysfunction in a dose-dependent manner. Global gene expression profiling revealed altered levels of the immediate early stress response genes (*Egr1*, *Egr2* and *Fos*), ß-myosin heavy chain (*Myh7*), and skeletal muscle actin (*Acta1*). We verified the effect of miR-499 on the immediate early response genes by miR-499 gain- and loss-of-function in vitro. Consistent with a role for miR-499 in blunting the response to cardiac stress, asymptomatic miR-499-expressing mice had an impaired response to pressure overload and accentuated cardiac dysfunction.

**Conclusions:**

Elevated miR-499 levels affect cardiac gene expression and predispose to cardiac stress-induced dysfunction. miR-499 may titrate the cardiac response to stress in part by regulating the immediate early gene response.

## Introduction

Heart failure affects over five million individuals in the United States and is characterized by progressive cardiac dysfunction. Such cardiomyopathies are characterized by significant changes in gene expression [1]–[7], many of which represent adaptive or maladaptive responses to stress. The immediate early gene response is activated rapidly upon cardiac stress and in a number of models of cardiac failure [8]. These genes are rapidly upregulated in response to environmental stimuli [9]–[11] and include the early growth response gene *Egr1* and the protooncogene *Fos*, both of which are involved in the cardiac stress response [12]–[14]. The induction of these genes is dependent upon serum response factor (SRF) and mediates further changes in gene expression, including regulation of sarcomeric and hypertrophic genes [15], [16].

MicroRNAs are small non-coding RNAs that play an important role in gene regulation. Each of the over 650 known human microRNAs likely regulates hundreds of mRNAs and thus has the potential to affect many biologic processes, although the effect on rapid transcriptional responses has not been extensively studied. Some microRNAs are expressed ubiquitously while others display tissue-specific expression patterns, suggesting unique functions within tissues. Several microRNAs, including miR-1, miR-133, miR-206 and miR-208 [17]–[29], are found in cardiac and/or skeletal muscle, and each has a potentially distinct regulatory function. Furthermore, many microRNAs reside within introns of coding genes, suggesting that their function may have co-evolved with their host gene [30], [31].

In screening for microRNAs enriched in the human heart, we identified an abundant microRNA, miR-499, which has been the subject of several recent studies. Global microRNA expression profiling studies have identified miR-499 in the heart [32]–[35], however its function is just beginning to be elucidated. miR-499 is an evolutionarily conserved muscle-specific microRNA that is encoded within the intron of myosin heavy chain 7B (*Myh7B*) and is highly enriched in the cardiac ventricles. miR-499 plays a role in myosin gene regulation [36]–[38], however the functional effects of altered microRNA dosage may depend on the tissue's physiologic state. Furthermore, microRNA levels vary in many cardiac disease states [30], [39]–[41], and in cardiac samples from individuals with aortic stenosis leading to pressure-overload and heart failure, miR-499 levels are altered [40]. In this study we show that elevated levels of miR-499 in hearts of transgenic mice result in cardiomyocyte hypertrophy and stress-dependent cardiac dysfunction. Furthermore, we found miR-499 alters the immediate early gene response to cardiac stress, which may partially contribute to the effects of elevated miR-499.

## Results

### miR-499 is expressed in human heart and skeletal muscle

**Figure 1:**
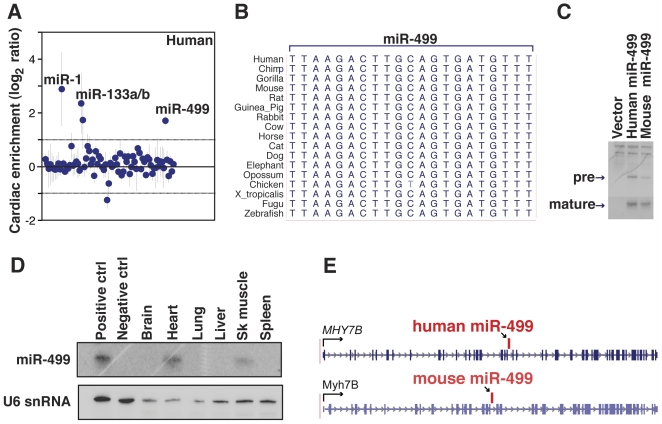
Human miR-499 is a conserved muscle-specific microRNA. (A) microRNA expression in human fetal heart compared to liver. Positive log_2_ ratio values indicate cardiac enrichment. n = 3 independent RNA samples per group, dots with vertical line indicate mean ± standard deviation for each microRNA. (B) Alignment of genomic sequences corresponding to mature miR-499 in multiple species. (C) Northern blot with a miR-499 specific probe with RNA from 293T cells transfected with the genomic sequence surrounding the human or mouse miR-499 locus. Pre-miR and mature microRNA bands are visible. (D) Mature miR-499 was expressed in human heart and skeletal muscle as detected by Northern blot; U6 small nuclear RNA was probed as a loading control. RNA from miR-499 transfected 293T cells or control vector-transfected cells was used as positive or negative controls (ctrl). (E) Genomic organization of miR-499 in the myosin heavy chain gene of human *MYH7B* or mouse *Myh7b* adapted from the UCSC genome browser. The conserved intronic location of miR-499 is indicated between exons 20 and 21 in humans and exons 19 and 20 in mouse; arrows indicate the direction of transcription.

To identify microRNAs that are enriched in the human heart, we compared the expression of microRNAs in human fetal heart and fetal liver using microRNA microarrays. miR-499 was among the top cardiac-enriched microRNAs (**Fig. 1A** **, Table S1**), along with the well-studied microRNAs, miR-1 and miR-133. miR-499 is distinct from miR-1 and miR-133 in that it is encoded in only one genomic locus. By genomic sequence alignment, miR-499 is completely conserved throughout evolution with the exception of a single nucleotide change in chicken (**Fig. 1B**). We verified that the predicted human miR-499 genomic region encoded the microRNA by cloning the pre-miR-499 region, expressing it in cell culture, and performing Northern blot analysis (**Fig. 1C**). To determine the tissue-specificity of miR-499, we surveyed various human tissue types by Northern blot; miR-499 was detected only in the heart and in skeletal muscle (**Fig. 1D**) in agreement with what has been reported in mice [36], [37]. *In silico* analysis revealed that miR-499 was located in an intron of the human myosin heavy chain gene, *MYH7B* (**Fig. 1E**), analogous to the arrangement in *Myh7B* in mice.

### Elevated miR-499 levels are associated with cardiac hypertrophy *in vivo*

**Figure 2:**
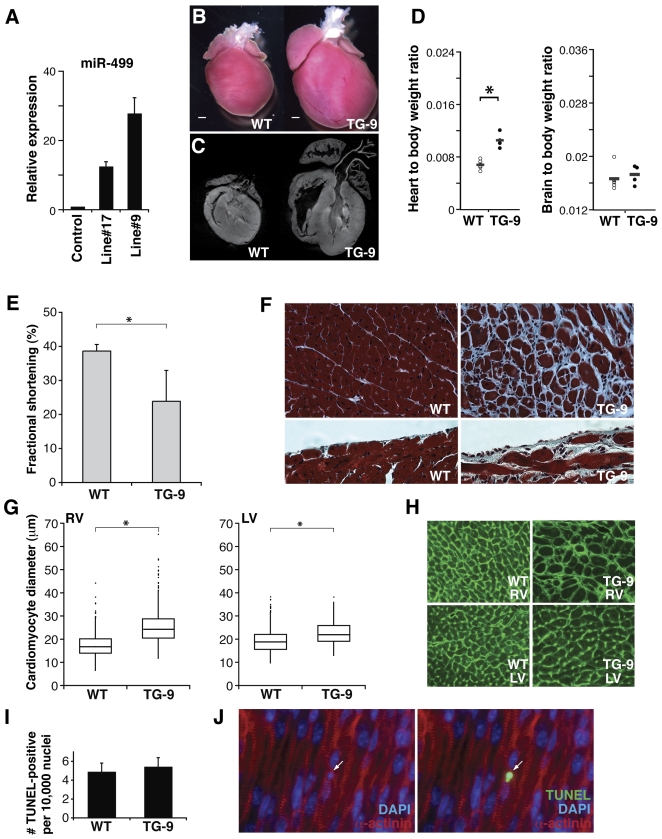
Characterization of hypertrophy phenotype in miR-499 transgenic mice. (A) Cardiac miR-499 expression levels (mean ± standard deviation) in transgenic mouse lines compared to littermate controls. (B) Hearts from miR-499 transgenic mice (line #9, TG-9) were larger than those from littermate controls (6 weeks of age), n = 16 mice per group. (C) Coronal optical tomography section of miR-499 TG-9 heart demonstrated chamber enlargement compared to control. (D) Heart-to-body weight ratios of miR-499 transgenic (TG-9) or littermate control (WT) mice. Brain-to-body weight ratios are also shown. Data from individual mice are plotted as dots with the mean indicated by a horizontal bar. *P = 0.0002. (E) Fractional shortening measured by echocardiography of WT or TG-9 mice. *P = 0.00047, n = 8 mice for WT, 4 for TG. (F) Histological section of heart revealing area of fibrosis in TG-9 hearts compared to WT as shown by Masson trichrome staining in myocardium (upper panels) or beneath the endocardial surface (lower panels); magnification 400×. (G) Quantification of cardiomyocyte size from TG-9 mice compared to WT mice (RV, right ventricle, and LV, left ventricle, both *P<0.05; box plots depicts size distribution of over 300 cells per heart in each of two mice per group). (H) Wheat germ agglutinin (WGA) staining; magnification 200×. (I) TG-9 and WT heart demonstrated similar levels of apoptosis, n = 3 mice per group; representative stained histologic section (J) demonstrated the alpha-actinin staining (red) and DAPI-stained nuclei (blue) overlaid with TUNEL-positive nuclei (green, right panel).

We hypothesized that miR-499 expression may be playing an important role in cardiac gene regulation given its expression pattern, its evolutionary conservation, and its decrement in pressure-overload conditions such as human aortic stenosis [40]. To determine the consequence of persistent miR-499 expression in pressure-overload stress, we first generated transgenic mice with increased levels of miR-499 in the heart by expressing miR-499 under control of the cardiac, alpha-myosin heavy chain (Myh6) promoter, as it confers increasing postnatal ventricular cardiomyocyte expression [42]–[44]. We established two transgenic lines, which varied in degree of miR-499 expression compared to littermate controls (**Fig. 2A**). Mice expressing lower levels of miR-499 (line #17 or TG-17) appeared normal under basal conditions (n = 20). Transgenic mice expressing higher levels of miR-499 (line #9 or TG-9) developed enlarged hearts (n = 16) as demonstrated by gross pathology at 5 weeks of life (**Fig. 2B**). The higher expressing miR-499 transgenic mice (TG-9) had larger hearts (**Fig. 2C**) and increased heart-to-body weight ratios compared to littermate controls (**Fig. 2D**), while the brain-to-body weight ratios were similar. Echocardiography demonstrated decreased fractional shortening in TG-9 mice indicating contractile dysfunction (**Fig. 2E** **,** **Table 1**).

**Table 1:** Echo Assessment of miR-499 Transgenic Mice.

	WT	TG-9	P value	WT	TG-17	P value	WT†	TG-17†	P value	WT TAB‡	TG-17 TAB‡	P value
%FS	38.63±1.95	23.88±9.06	**0.00047** *	40.67±0.88	37.42±4.82	0.31	39.7±4.16	36.3±4.13	0.34	30.9±2.29	19.9±4.10	**0.00079** *
LVEDD	3.85±0.18	4.18±1.06	0.37	3.71±0.23	3.83±0.09	0.38	3.79±0.41	3.81±0.11	0.95	3.74±0.08	4.18±0.27	**0.0095** *
LVESD	2.37±0.17	3.24±1.21	**0.047** *	2.20±0.14	2.40±0.24	0.26	2.29±0.37	2.43±0.21	0.57	2.58±0.09	3.35±0.38	**0.0023** *
LVPWd	0.72±0.05	0.76±0.11	0.43	0.72±0.08	0.68±0.03	0.42	0.79±0.07	0.81±0.04	0.66	1.08±0.03	1.05±0.04	0.26
LVPWs	1.20±0.07	1.00±0.15	**0.0054** *	1.19±0.06	1.12±0.08	0.25	1.31±0.06	1.26±0.09	0.44	1.46±0.09	1.23±0.11	**0.0078** *

Measurements of left ventricle (LV) in mm ± standard deviation, LVEDD, end-diastolic dimension; LVESD, end-systolic dimension; LVPWd, posterior wall diastole; LVPWs, posterior wall systole. n = 8 for WT, 4 for TG-9, 3–5 for TG-17. P values calculated using student's T-test.

† Second assessment 5 weeks later.

‡ TAB 3.5 mo after thoracic aortic banding.

*P<0.05.

On histopathological analysis, we found extensive cellular hypertrophy and patchy interstitial fibrosis in TG-9 hearts (**Fig. 2F**) (n = 4). To assess the degree of hypertrophy, we quantified cardiomyocyte cross-sectional area after staining cardiac sections using wheat germ agglutinin (WGA). TG-9 hearts displayed a marked increase in cardiomyocyte size indicating cellular hypertrophy (**Fig. 2G and 2H**). There was no increase in TUNEL-positive/alpha-actinin-positive cells in TG-9 mice at 1 week (**Fig. 2I and J**) or 7 weeks of age, suggesting that cardiomyocyte apoptosis was not a primary contributor to the phenotype.

**Figure 3:**
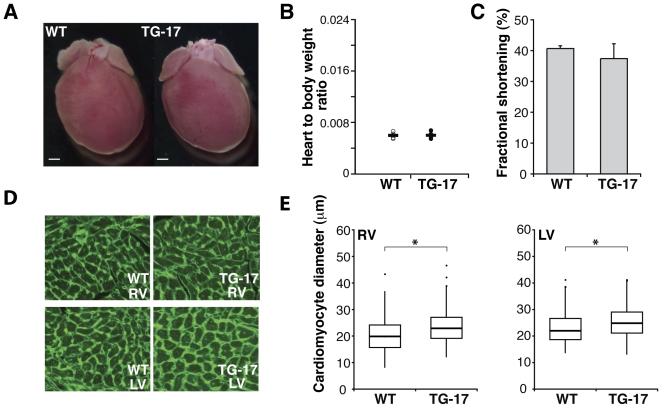
Modest levels of miR-499 expression results in mild hypertrophy. (A) Gross appearance of hearts from miR-499 transgenic mice (line #17, TG-17) and littermate controls (WT) was similar. (B) Heart-to-body weight ratios in TG-17 and WT, n = 4–6 mice per group. (C) Fractional shortening of WT or TG-17 mice by echocardiography. (D) WGA staining of histological sections of hearts from TG-17 and WT mice; magnification 200×. (E) Quantification of cardiomyocyte size revealed an increase in TG-17 (RV and LV both *P<0.05).

A second line of miR-499 transgenic mice, expressing relatively lower levels of miR-499 (line #17, TG-17), had hearts that appeared unremarkable under basal conditions (**Fig. 3A**). The ratio of the heart-to-body weights was similar in transgenic and control mice (**Fig. 3B**), and echocardiographic evaluation of cardiac function in TG-17 mice trended lower, although the results were not statistically significant, at least under basal conditions (**Fig. 3C** **,** **Table 1**). However, WGA-staining of TG-17 hearts revealed a mild but significant increase in cardiomyocyte size (**Fig. 3D and E**), indicating mild cellular hypertrophy.

### miR-499 targeting of Sox6

**Figure 4:**
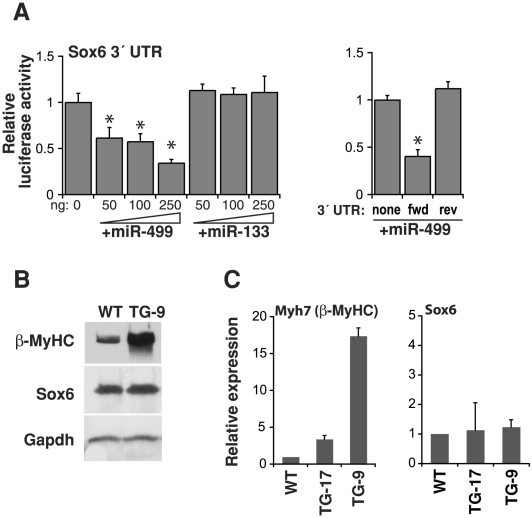
miR-499 targeting of Sox6. (A) The 3′UTR of Sox6 was placed downstream of a luciferase reporter construct and tested for repression by miR-499 in 293T cells. Sox6 3′UTR-mediated repression increased as amounts of miR-499 was increased; this was not observed with miR-133 or when the UTR orientation was reversed, n = 3–4 transfections per condition, *P<0.05. (B) Western blots indicate protein levels in TG-9 or WT hearts using antibodies against Sox6, ß-myosin heavy chain (ß-MyHC), or Gapdh. Results are representative of three hearts each. (C) Cardiac expression of ß-myosin heavy chain (ß-MyHC) or Sox6 mRNA transcripts in TG lines and WT mice by qPCR, n = 3.

Given the baseline hypertrophy in miR-499-transgenic mice, we hypothesized that miR-499 expression may alter genes important in this cellular program, either directly or indirectly. Initially, we examined genes predicted to be targets of miR-499 using a targeted approach. From a survey of microRNA target prediction programs including MiRanda, TargetScan, and Mirtarget, Sox6 emerged as an attractive candidate, given three potential miR-499 binding sites and potential roles for Sox6 in muscle and heart [36]–[38], [45], [46]. We verified that Sox6 was an *in vitro* target of miR-499 by cloning the Sox6 3′-UTR and then tested for microRNA repression by luciferase assays. We transfected increasing amounts of miR-499 into 293T cells in culture and found dose-dependent inhibition of the Sox6 UTR-luciferase construct, however another cardiac microRNA, miR-133, had no effect regardless of the dose (**Fig. 4A**). Surprisingly, Sox6 protein levels were not diminished in transgenic mice, however the myosin heavy chain classically involved in hypertrophy, ß-myosin heavy chain (ß-MyHC), was markedly increased (**Fig. 4B**). This was accompanied by an increase in the corresponding *Myh7* (ß-MyHC) transcript, while Sox6 mRNA was unaltered, similar to its protein levels (**Fig. 4C**).

### A distinctive pattern of cardiac gene regulation mediated by miR-499

**Figure 5:**
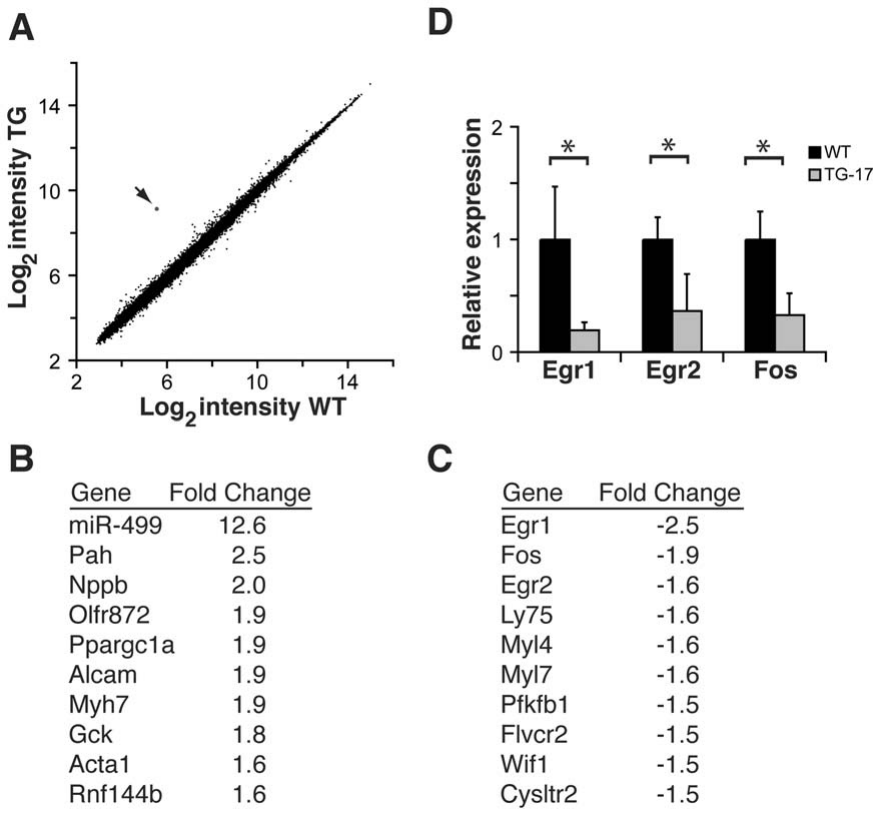
Altered cardiac transcripts from global analysis of miR-499 TG hearts. (A) Expression microarray results depicting average log_2_ intensity of gene expression from post-natal day 17 WT hearts (x-axis) versus TG-17 hearts (y-axis). Each dot represents a single transcript from three miR-499 transgenic mice and three littermate controls. The dot indicated in red (arrow) represents miR-499 expression. (B) and (C) Gene expression alterations in miR-499 mice (top ten up- and downregulated genes). Egr1 and Fos were the most downregulated transcripts. Full expression data have been deposited in NCBI GEO. (D) qPCR validation of microarray results regarding the immediate early response gene transcripts Egr1, Egr2, and Fos. *P<0.05, n = 3 hearts per group with samples assessed in triplicate. Bars indicate mean with 95% confidence interval.

We investigated miR-499-dependent changes in cardiac gene expression by comparing global gene expression in miR-499-transgenic mice and littermate controls. To avoid changes secondary to heart failure, we used the miR-499 transgenic line (TG-17) that did not display overt cardiac dysfunction. RNA was prepared from the ventricles of postnatal day 17 miR-499 transgenic mice or littermate controls, and gene expression was compared by microarray analysis (**Fig. 5A**). When we analyzed for differentially expressed genes, 0.03% of the transcripts (nine transcripts of the 28,853 interrogated) were dysregulated when we used a stringent cut-off (P value<0.05, Benjamini-Hochberg correction for false-discovery rate), whereas a less stringent cut-off demonstrated that 0.5% of the transcripts (172 transcripts of the 28,853 interrogated) were significantly altered (Bayes log odds of differential expression B>0). The average fold change was 1.43 for upregulated genes and −1.37 for downregulated genes with a range of 2.51 to −2.53 fold. miR-499 levels were increased 12.6-fold, in agreement with our microRNA qPCR results.

Transcripts such as *natriuretic peptide precursor type B* (*Nppb*), *ß-myosin heavy chain* (*Myh7*), and *alpha 1 skeletal muscle actin* (*Acta1*), genes that are known to be upregulated in hypertrophy, were increased 2.0, 1.9, and 1.6 fold, respectively (**Fig. 5B**). *Sox6*, which was a target for miR-499 in luciferase assays, was not altered in transgenic mice (1.0 fold, not significant). We noted that the most downregulated transcripts were the immediate early response genes, *early growth response 1* (*Egr1*) (−2.5 fold, the most downregulated transcript in the entire array) and *FBJ osteoscarcoma oncogene* (*Fos*) (−1.9 fold, the second most downregulated transcript) (**Fig. 5C**). *Egr2*, an immediate early gene with an unknown role in the heart, was diminished −1.6 fold, and was also among the ten most downregulated transcripts. As the immediate early genes may be involved in the response to stress, we validated the reduced *Egr1*, *Egr2*, and *Fos* levels using qPCR, which further suggested dysregulation (**Fig. 5D**).

### The Immediate Early Gene Response is Altered by miR-499 Levels

**Figure 6:**
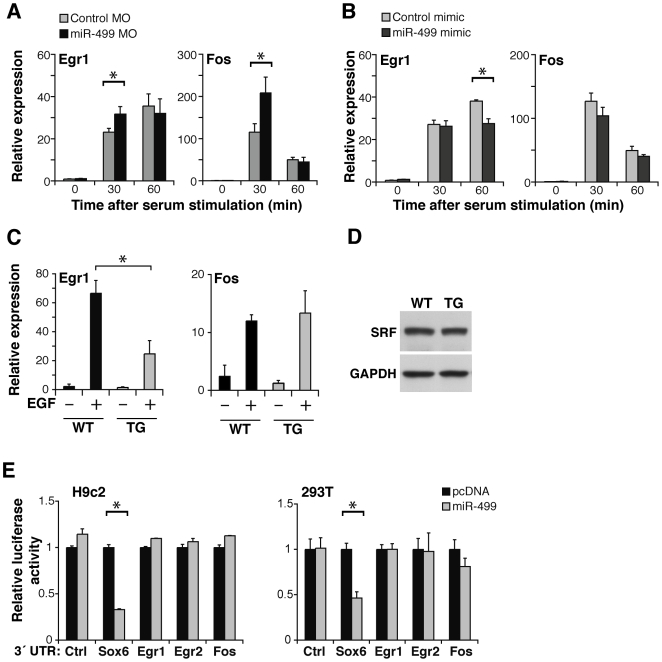
miR-499 blunts the induction of the immediate early response genes. (A) Egr1 and Fos mRNA levels by qPCR relative to Gapdh in the ventricular cell line H9c2, upon introduction of a morpholino (MO) that blocks miR-499 generation or a control MO. (B) Egr1 and Fos mRNA levels by qPCR in H9c2 cells after the introduction of miR-499 or control mimic. The x-axis in (A) and (B) indicates the time after serum stimulation to activate the immediate early gene response; n = 3 per group. *P<0.05. (C) Egr1 or Fos mRNA levels in miR-499 TG-17 or WT hearts as assessed by qPCR 1.5 hours after *in vivo* stimulation with epidermal growth factor (EGF). Bars indicate mean ± standard deviation; n>3 per group. *P<0.05. (D) Western blot indicates protein levels in TG-9 or WT hearts using SRF antibodies with Gapdh as loading control. Results are representative of three hearts each. (E) The 3′ UTR of Sox6, Egr1, Egr2, or Fos was placed downstream of a luciferase reporter construct and tested for repression by miR-499 in culture. Sox6 3′UTR-mediated repression by miR-499 was evident, however Egr1, Egr2, and Fos 3′UTRs were not repressed by miR-499 (n = 3 per condition, *P<0.05).

We reasoned that the immediate early response genes may be playing a role in the hypertrophic phenotype for several reasons: 1) the immediate early response genes are rapidly altered in response to cardiac stress [8], [47], [48]; 2) perturbation of the immediate early response alters cardiac hypertrophy [49], [50]; and 3) immediate early genes are typically SRF-dependent, and the cardiac abnormalities in the miR-499 transgenic partially resembled that of mice where SRF was temporally deleted [51]. First, to examine the miR-499:immediate early gene relationship independent of the potential complexity of the *in vivo* transgenic system, we tested whether the immediate early gene response was altered using cultured cells where miR-499 levels were manipulated. We used an antisense morpholino to inhibit miR-499 generation in the ventricular cardiomyocyte line, H9c2. Knockdown of miR-499 levels led to an increase in Egr1 and Fos levels 30 min following immediate early gene response activation by serum (**Fig. 6A**), however the effect was temporally limited, as it resolved by 60 min. Conversely, introduction of miR-499 mimic lowered Egr1 levels in cell culture after serum stimulation (**Fig. 6B**). There was no significant effect on Fos in this setting. These data are consistent with a role for miR-499 in regulating the immediate early gene response.

We next investigated whether elevated miR-499 levels affected the epidermal growth factor (EGF)-induced immediate early gene response *in vivo* [52]. miR-499-transgenic mice (TG-17) displayed a blunted activation of Egr1 in response to systemic administration of EGF (**Fig. 6C**). Activation of Fos was not different in the miR-499 transgenic mice, similar to what we observed in cultured cells. These data suggested that miR-499 levels negatively regulate the stress-induced activation of Egr1 and this may be important in the cardiac response to stress and in hypertrophy.

We assessed whether serum response factor (SRF), which is upstream of the immediate early genes, was altered in the miR-499 transgenics. When we compared SRF protein levels in WT and TG mice, there was no difference (**Fig. 6D**), suggesting that the immediate early genes are regulated by miR-499 independent of SRF levels. To test whether miR-499 directly or indirectly regulates the immediate early genes, we cloned the 3′ UTRs of the immediate early response genes and placed each downstream of luciferase and tested these in H9c2 and 293T cells. miR-499 did not repress luciferase constructs with the Egr1, Egr2, or Fos UTRs (**Fig. 6E**), whereas miR-499 repressed constructs containing the Sox6 3′UTR. Since the 3′ UTRs of Egr1, Egr2, and Fos were insufficient to mediate miR-499 repression, we suspect they are affected by miR-499 in a yet unknown manner that is not dependent on altered SRF levels.

### miR-499 Expressing Mice Are Predisposed to Stress-Induced Cardiac Dysfunction

**Figure 7:**
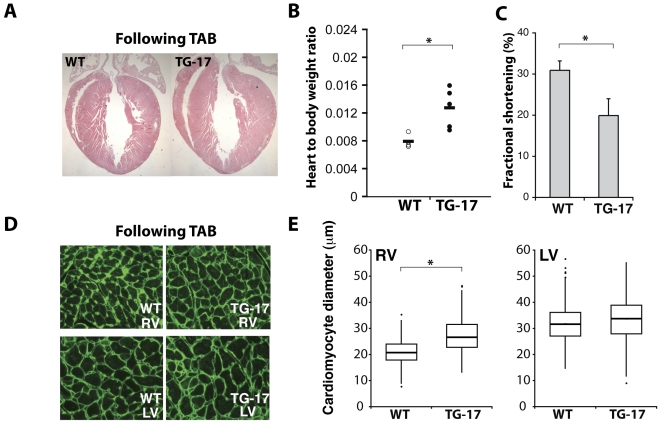
miR-499 elevation predisposes to stress-induced cardiac dysfunction in vivo. (A) Following thoracic aortic banding (TAB), TG-17 hearts were slightly larger compared to banded WT hearts, and (B) the heart-to-body weight ratio was increased to a greater degree in TG-17 compared to similarly banded WT controls (n = 4–5 per group, *P<0.05). (C) Fractional shortening of WT and TG-17 hearts by echocardiography, *P = 0.00079. (D) WGA staining and quantification revealed increased size of cardiomyocytes following TAB. *P<0.05.

Given the effect of miR-499 levels on the immediate early gene response, which is known to be involved in cardiac stress and hypertrophy [12]–[14], we hypothesized that increased miR-499 levels may predispose to stress-induced cardiac dysfunction. We therefore tested whether the TG-17 miR-499-transgenic mice, which had normal function at baseline, were predisposed to increased cardiac dysfunction upon cardiac pressure overload. We used the well-characterized model of thoracic aortic banding (TAB) to induce pressure overload in miR-499 transgenic (TG-17) or control mice and monitored cardiac function over 12 weeks. The banded transgenic hearts demonstrated accentuated cardiac enlargement (**Fig. 7A**) and a marked increase in heart weight to body weight ratio (**Fig. 7B**) compared to banded WT controls. After TAB, the miR-499 transgenic mice displayed more severe contractile dysfunction as indicated by the decline in fractional shortening compared to control mice (**Fig. 7C** **,** **Table 1**) that underwent the same degree of TAB (peak aortic pressure gradient: WT 49±10 mmHg, TG 43±9 mmHg, p = 0.251). Interestingly, the cardiomyocyte size was not higher in the LV, but was slightly increased in the RV of transgenic mice (**Fig. 7D and 7E**), similar to TG-9 mice. Overall, these results suggest elevated miR-499 levels predispose the heart to cardiac dysfunction.

To determine the dose-responsiveness of miR-499 sensitive genes, we analyzed gene expression in the higher miR-499 expressing TG-9 line at 17 days of age. Thirty-two common transcripts were altered in both TG-17 and TG-9, and thirty of these were greater in magnitude in TG-9 than TG-17 (**Table S2**). None of the genes were predicted by Targetscan. Importantly, pathway analysis revealed enrichment for the GO terms sarcomere (P = 0.0074), contractile fiber part (P = 0.0085), myofibril (P = 0.0095), and contractile fiber (P = 0.010), further highlighting the potential role of miR-499 in regulating sarcomeric function in the heart.

## Discussion

In this study, we found that elevated levels of miR-499 in the heart can lead to cardiomyocyte hypertrophy and cardiomyopathy in a dose-dependent manner. High levels of miR-499 led to spontaneous cardiac contractile dysfunction, while more modest levels of transgenic expression conferred susceptibility to pressure-induced dysfunction.

Our studies support an association between elevated cardiac miR-499 levels and cardiac dysfunction, particularly in the setting of pressure overload. Interestingly, in a study that analyzed microRNAs in human aortic valve stenosis, miR-499 levels were decreased [40], and it is possible that this decrement may regulate cardiac gene expression changes that are important in the physiologic response to cardiac pressure load. We suspect the failure of normal miR-499 down-regulation in our transgenic mice disrupted the normal response to cardiac pressure stress; similar theories have recently been put forward for cardiac transgenic mice expressing miR-133a [53]. Interestingly, a recent study [39] reported that miR-499 levels increase in human cardiac failure, and the findings in our transgenic mouse model may support a detrimental role for elevated miR-499 levels.

We identified a discrete set of altered transcripts in unmanipulated, normally functioning, miR-499 transgenic hearts, including natriuretic peptide precursor type B (*Nppb*), ß-MyHC (*Myh7*) and alpha 1 skeletal muscle actin (*Acta1*). This suggests gene expression changes due to miR-499 that favor hypertrophy. These changes were confirmed in the second transgenic line. The limited magnitude of transcript changes by miR-499 suggests that the hypertrophic phenotype observed was not due to gross gene expression disruption. Although the Sox6 3′UTR could be targeted by miR-499, as we and others have demonstrated [36]–[38], [46], Sox6 levels were not altered in the transgenic mice, suggesting that Sox6 was not targeted by increased miR-499 levels *in vivo*, or that additional compensatory mechanisms may be involved *in vivo*. It is possible that targets seen in luciferase assays may not necessarily translate into targeting *in vivo*.

We present several lines of evidence that demonstrate that miR-499 levels fundamentally alter the immediate early gene response, which is known to be important in the cardiac stress response. First, Egr1 and Fos were the most diminished transcripts in TG-17 miR-499-altered hearts, suggesting a robust alteration in the immediate early gene expression program, while in TG-9 Egr1 was also altered (although Fos was not significantly changed). Second, inhibition of miR-499 in culture led to increases in induced Egr1 and Fos levels, which demonstrates that this regulation is present even outside of the context of the cardiac transgene. Pressure-induced stress, which is known to invoke the immediate early gene response, dramatically altered cardiac function in transgenic mouse hearts. We therefore hypothesize that miR-499 alters the cardiac response to stress in part by modulating the immediate early gene response.

The connection between miR-499 and the immediate early gene response is important since activation of this pathway is thought to precede further transcriptional responses to stress. Similar to TG-17 mice expressing miR-499, mice deficient in Egr1 are normal at baseline, but have an impaired response to cardiac stress [49], as do transgenic mice expressing the Egr1-repressor, Nab1 [50]. Interestingly, mice lacking the cardiac-specific microRNA miR-208a have decreased amounts of miR-499 [25], [36]. Evaluation of genes reported to be dysregulated in miR-208a mutant mice revealed elevated levels of Egr1, Egr2, and Fos in the heart, suggesting a potential relationship between miR-499 and miR-208 in regulation of the immediate early gene response to cardiac stress. The observation that miR-499 transgenic mice develop or are predisposed to cardiac dysfunction may reflect the fact that the immediate early response genes hold a key position in the hierarchy of the cardiac transcriptional response to stress. It will be interesting to determine if levels of this non-coding RNA affect the progression of disease in the setting of pressure overload, such as in hypertension or aortic valve disease.

## Materials and Methods

### Ethics Statement

Care and handling of all experimental animals used in this study were in accordance with the University of California San Francisco's Institutional Animal Care and Use Committee policies and approved under protocol #AN080001.

### MicroRNA detection by Northern blotting

Total RNA (12 mcg) was separated on 12% polyacrylamide/urea gels and transferred to nitrocellulose membranes. Membranes were probed with ^32^P-labelled locked nucleic acid probe (Exiqon) or DNA oligonucleotide probe complementary to mature miR-499 or to U6. We transfected 293T cells (ATCC) with miR-499-expression construct or vector alone for RNA for positive and negative controls. Signals were detected by phosphoimaging.

### MicroRNA and messenger RNA detection by PCR

RNA samples were reverse transcribed for microRNA detection with microRNA-specific primers (Applied Biosystems) or for mRNAs with oligo-dT (Invitrogen). miR-499 levels and mRNAs were quantified using RNA from transgenic mouse hearts and littermate controls. Quantitative real-time PCR (qPCR) was performed in an ABI HT7900 with Taqman primers (Applied Biosystems). Taqman primers used: miR-499 (001352), miR-16 (000391), ß-myosin heavy chain (Myh7, Mm00600555_m1), Sox6 (Mm01274768_m1), Egr1 (Mm00656724_m1), Egr2 (Mm00456650_m1), Fos (Mm01302932_g1).

### MicroRNA genomic cloning

We amplified fragments containing miR-499 from genomic DNA using high-fidelity PCR (PlatinumTaq, Invitrogen) and directional cloning into pcDNA3.1-TOPO (Invitrogen) for expression studies in cell culture. The UCSC genome browser and Sanger miRBase were used to analyze cloned products, to assess microRNA conservation, and for primer design.

### MicroRNA target assays

We surveyed mutliple bioinformatics prediction programs that predict potential microRNA targets. MiRanda, TargetScan, and Mirtarget [54]–[56] included Sox6 as a potential target. The 3′ untranslated regions (UTRs) of Sox6, Egr1, Egr2, or Fos were cloned into pcR2.1-TOPO (Invitrogen) and subcloned downstream of the luciferase coding sequence in pMIR-REPORT (Ambion). For microRNA target assays, we transfected cells with microRNA expression vector (50, 100, and 250 ng) and luciferase construct containing the target sequence of interest. Briefly, we expressed microRNAs in H9c2 or 293T cells with expression plasmids, equalized the total amount of DNA transfected, and expressed the luciferase construct with the 3′ UTR of interest (Lipofectamine 2000, Invitrogen). We also tested whether repression was lost when we transfected the reversed 3′UTR sequence construct with our miR-499 vector (250 ng). Renilla luciferase-expressing plasmid (pRL-CMV, Promega) was used to normalize transfection efficiency.

### Transgenic mice

To express miR-499 *in vivo*, we amplified DNA surrounding the mouse pre-miR-499 region and cloned the 574-bp fragment into the alpha-myosin heavy chain promoter vector using the following primers and SalI linkers: 5′-CGT GTC GAC CAA GTC TGG GGT GAA AGA GAA G-3′ (forward), 5′-TGT GTC GAC GGT CAT GAG CTT GTT GAG GTT C-3′ (reverse) and injected into one-cell FVB/NCrl embryos before implantation in pseudopregnant females. Phenotyping was performed by echocardiography (Vevo 770, VisualSonics) with standard measurement techniques (reviewed in [57]). Echocardiography was performed using anesthesia with isofluorane and with continuous monitoring of mice to ensure the heart rate was kept constant. Thoracic aortic banding was performed as described [58] with slight modifications. Briefly, the aorta was exposed through a mediastinal approach, and suture was tied around a 27-gauge needle placed adjacent to the aorta. Echocardiography confirmed aortic constriction by peak velocity measurement. For analysis of transgenic hearts, hearts were harvested in PBS containing 25 mM KCl and optical projection tomography was performed for virtual sectioning [59]. Histologic analysis was performed using Masson Trichrome staining, DAPI, alpha-actinin staining (clone EA-53, Sigma), and wheat germ agglutinin staining on corresponding coronal sections followed by quantification of cardiomyocyte cross-sectional area by measuring the cell diameter with visible nuclei in over 300 cells. Cell death was assessed by quantification using alpha actinin-staining combined with tunel analysis (Roche) at different ages. For analysis of the immediate early gene response *in vivo*, recombinant epidermal growth factor (30 mcg, Invitrogen) or vehicle (normal saline) was injected intraperitoneally into control or transgenic mice, and hearts were collected after 1.5 hours for analysis of immediate early gene expression.

### Western blotting

Protein lysates were run on SDS-PAGE gels, transferred to PVDF membranes, and probed with antibodies to Sox6 (ab12054, Abcam), slow myosin (clone NOQ7.5.4D, Sigma), SRF (sc-335, Santa Cruz), or Gapdh (Santa Cruz).

### Gene expression analysis

Data have been deposited in The National Center for Biotechnology Information (NCBI) Gene Expression Omnibus (GEO, http://www.ncbi.nlm.nih.gov/geo/) series accession number GSE21104 (http://www.ncbi.nlm.nih.gov/geo/query/acc.cgi?token=nrqpfuaaqeacsxo&acc=GSE21104). Total RNA was isolated from ventricular tissue from three miR-499-transgenic mice (line #17, TG-17) and three littermate controls at postnatal day 17. Gene expression was assessed using Mouse Gene 1.0 ST Array as recommended by the manufacturer (Affymetrix). Differentially expressed genes were identified using the limma package. Adjustment for multiple testing was performed as described in the text. Further validation was performed using qPCR as described above. Human heart and liver RNA samples (Virogen), gestational age 6–8 weeks, were labeled and run for microRNA expression as described by the manufacturer (Exiqon). Gene ontology analysis was performed using DAVID Bioinformatics Resources 6.7 [60], [61].

### MicroRNA modulation in cell culture

To modulate microRNA levels, miR-499 mimic or control mimic (Dharmacon) or a morpholino directed against the miR-499 precursor or a scrambled control morpholino (GeneTools) was electroporated (Amaxa) into H9c2 and allowed to recover overnight. After serum starvation in 1% fetal bovine serum, 10% serum was applied for designated times and RNA collected for RT-PCR analysis. Sequence of morpholino directed against the miR-499 precursor 5′-ATG CAG AGG AGC TAA ACA TCA CTG C-3′ or a scrambled control morpholino 5′-GAT TGC GAA GAG TCT CAC AAG ACC A-3′.

### RNA isolation

Total RNA was isolated using guanidine thiocyanate and phenol (Molecular Research Center, Inc.) and following manufacturer's instructions for chloroform extraction, isopropanol precipitation, and 75% ethanol washing. For tissue RNA extraction, tissues were processed using a bead homogenizer (WisBiomed).

### Primer sequences

Genomic amplification primers for human miR-499: 5′-CAC CCA AGT CTG GGG TGA AAG AGA AG-3′ (forward) and 5′-GGT CAT CAG CTT GTT GAG GTT C-3′ (reverse). 3′UTR sequence primers: Egr1 5′-GGC AGG AAA GAC ATA AAA GCA C-3′ (forward) and 5′-ACA TAT CCC ATG GGC AAT AGA G-3′ (reverse), Egr2 5′-AAC ACT ACC ACC CTT CCC TGT T-3′ (forward) and 5′-AGC CAT CCA TTA TCT GAA CTC C-3′ (reverse), Fos 5′-TGG TGC ATT ACA GAG AGG AGA A-3′ (forward) and 5′-TGG AAC AAT AAG CAA ACA ATG C-3′ (reverse), Sox6 5′-CTC ACT AGT TGG CTC CAC AAT ACA TCA GC-3′ (forward) and 5′-CTC AAG CTT CCA AGT GAC AAA ATG GCT CA-3′ (reverse).

## Supporting Information

Table S1 Enrichment of microRNAs in human heart versus liver. Log ratio for each microRNA and standard deviation are shown. Positive ratios indicate enrichment in heart compared to the liver.
(DOC)

Table S2 Common genes altered in miR-499 transgenic lines, TG-17 and TG-9. For each gene, the gene symbol, genomic location, log ratios and fold change of TG versus WT are shown.
(XLS)
