# Supplementary material for: Elevated miR-499 Levels Blunt the Cardiac Stress Response
Source: PLoS One. 2011 May 9;6(5):e19481. doi: 10.1371/journal.pone.0019481 (PMC3090396; doi:10.1371/journal.pone.0019481)
Supplement: Table S1 — Enrichment of microRNAs in human heart versus liver. Log ratio for each microRNA and standard deviation are shown. Positive ratios indicate enrichment in heart compared to the liver. (DOC) [file pone.0019481.s001.doc]

**Table S1. Enrichment of microRNAs in human heart versus liver**

| ID | Standard deviation | Log ratio |
| --- | --- | --- |
| hsa-miR-1 | 1.37 | 2.79 |
| hsa-miR-133a-133b | 1.12 | 2.26 |
| hsa-miR-133b | 1.09 | 1.64 |
| hsa-miR-499 | 0.17 | 1.62 |
| hsa-miR-122a | 0.49 | 0.67 |
| hsa-miR-143 | 0.44 | 0.67 |
| hsa-miR-30b | 0.07 | 0.59 |
| hsa-miR-145 | 0.54 | 0.49 |
| hsa-miR-184 | 0.19 | 0.48 |
| hsa-miR-30c | 0.27 | 0.42 |
| hsa-miR-22 | 0.50 | 0.40 |
| hsa-miR-27a | 0.13 | 0.30 |
| hsa-miR-197 | 0.11 | 0.29 |
| hsa-miR-24 | 0.19 | 0.28 |
| hsa-miR-23a | 0.20 | 0.28 |
| hsa-miR-346 | 0.19 | 0.25 |
| hsa-miR-21 | 0.33 | 0.23 |
| hsa-miR-142-3p | 0.53 | 0.21 |
| hsa-miR-324-5p | 0.03 | 0.20 |
| hsa-miR-198 | 0.15 | 0.20 |
| bta-miR-140 | 0.18 | 0.18 |
| hsa-miR-365 | 0.55 | 0.18 |
| hsa-miR-16 | 0.19 | 0.17 |
| hsa-miR-503 | 0.33 | 0.16 |
| hsa-miR-451 | 0.81 | 0.15 |
| hsa-miR-513 | 0.20 | 0.10 |
| hsa-miR-483 | 0.55 | 0.10 |
| hsa-miR-126 | 0.23 | 0.10 |
| hsa-miR-23b | 0.29 | 0.09 |
| hsa-miR-326 | 0.18 | 0.09 |
| hsa-miR-27b | 0.15 | 0.09 |
| hsa-miR-492 | 0.57 | 0.08 |
| hsa-miR-202 | 0.24 | 0.04 |
| hsa-let-7g | 0.44 | 0.02 |
| hsa-miR-100 | 0.18 | 0.02 |
| hsa-miR-129 | 0.55 | 0.02 |
| gga-miR-140* | 0.21 | 0.01 |
| hsa-miR-185 | 0.21 | 0.00 |
| hsa-miR-125b | 0.39 | 0.00 |
| hsa-miR-99b | 0.07 | 0.00 |
| hsa-miR-15b | 0.23 | 0.00 |
| hsa-miR-212 | 0.06 | -0.01 |
| hsa-let-7b | 0.77 | -0.01 |
| hsa-miR-199b | 0.46 | -0.01 |
| hsa-miR-107 | 0.19 | -0.04 |
| hsa-miR-370 | 0.17 | -0.04 |
| hsa-miR-20a | 0.21 | -0.05 |
| hsa-miR-125a | 0.40 | -0.05 |
| hsa-miR-452 | 0.19 | -0.07 |
| hsa-miR-20a | 0.23 | -0.10 |
| hsa-miR-136 | 0.35 | -0.11 |
| hsa-miR-26b | 0.37 | -0.11 |
| hsa-miR-199a | 0.09 | -0.11 |
| hsa-let-7f | 0.43 | -0.11 |
| hsa-let-7c | 0.53 | -0.13 |
| hsa-miR-106a | 0.20 | -0.13 |
| hsa-miR-451 | 0.76 | -0.14 |
| hsa-miR-498 | 0.72 | -0.15 |
| hsa-miR-376a | 0.05 | -0.15 |
| hsa-miR-106b | 0.23 | -0.16 |
| hsa-let-7d | 0.47 | -0.17 |
| hsa-let-7a | 0.69 | -0.17 |
| hsa-miR-296 | 0.83 | -0.17 |
| hsa-miR-320 | 0.15 | -0.18 |
| hsa-let-7i | 0.25 | -0.19 |
| hsa-let-7e | 0.37 | -0.20 |
| hsa-miR-199a* | 0.31 | -0.21 |
| hsa-miR-130a | 0.20 | -0.24 |
| hsa-miR-92 | 0.25 | -0.27 |
| hsa-miR-222 | 0.24 | -0.28 |
| hsa-miR-101 | 0.10 | -0.28 |
| hsa-miR-214 | 0.11 | -0.28 |
| hsa-miR-373* | 0.25 | -0.36 |
| hsa-miR-26a | 0.45 | -0.37 |
| hsa-miR-335 | 0.15 | -0.40 |
| hsa-miR-221 | 0.18 | -0.41 |
| hsa-miR-17-5p | 0.28 | -0.43 |
| hsa-miR-512-5p | 0.24 | -0.44 |
| hsa-miR-200b | 0.46 | -0.72 |
| hsa-miR-200a | 0.30 | -1.33 |
